# Knowledge Structure and Evolution of Hotspots in Play Therapy: A Bibliometric Analysis via Multiple Visualization Tools

**DOI:** 10.1002/brb3.71301

**Published:** 2026-03-10

**Authors:** Xinxing Fei, Shiqi Wang, Jianxiong Wang, Yaqian Gao, Yue Hu

**Affiliations:** ^1^ Department of Psychiatry Chengdu Eighth People's Hospital (Geriatric Hospital of Chengdu Medical College) Chengdu PR China; ^2^ Rehabilitation Medicine Center and Institute of Rehabilitation Medicine West China Hospital Sichuan University Chengdu Sichuan PR China; ^3^ Key Laboratory of Rehabilitation Medicine in Sichuan Province Chengdu Sichuan PR China; ^4^ Department of Rehabilitation Medicine The Affiliated Hospital of Southwest Medical University Luzhou PR China; ^5^ Rehabilitation Medicine and Engineering Key Laboratory of Luzhou Luzhou PR China; ^6^ Department of Rehabilitation Medicine The First Affiliated Hospital of Chengdu Medical College Chengdu PR China; ^7^ Centro Studi e Ricerche in Neuroscienze Cognitive, Dipartimento di Psicologia Alma Mater Studiorum – Università di Bologna, Campus di Cesena Cesena Italy

**Keywords:** bibliometrics, child care, cognitive behavioral therapy, play therapy, psychotherapy

## Abstract

**Background:**

Play therapy has seen growing clinical application and theoretical development, yet no dedicated bibliometric analysis has systematically mapped this field. This study provides the first systematic map of the global play therapy research landscape, identifying clinical trends and future priorities for the field.

**Methods:**

Relevant literature was retrieved from the Web of Science Core Collection, and bibliometric analyses were performed using Bibliometrix and VOSviewer.

**Results:**

A total of 771 articles were included. The United States led in publications, citations, international collaboration, and institutional participation. The *Arts in Psychotherapy* and Ray DC were the most productive journal and author, respectively. Core research focused on “children” and “adolescents,” while “autism,” “spectrum,” and expanded age‐group focus emerged as potential frontiers. However, a “visibility gap” remains, where clinical innovations from the Global South are often sidelined by Western‐centric indexing and credentialing systems.

**Conclusions:**

For clinicians, these findings underscore the need to adapt traditional techniques into neurodiversity‐sensitive and culturally grounded practices. For policymakers, the evidence supports integrating play therapy into national mental health guidelines and insurance frameworks as a cost‐effective, transdiagnostic intervention. Ultimately, bridging the gap between Western frameworks and regional adaptations is essential for creating a more inclusive and evidence‐based global mental health strategy.

## Graphical abstract

Our study analyzes the research status and evolution of play therapy through bibliometric and visualization analysis. Play therapy research has been growing steadily, and the United States leads in publications, citations, and international cooperation. There is an increasing focus on autism spectrum disorder and the inclusion of diverse age groups.

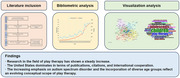

**Abbreviations**
APT: Association for Play Therapy
CCPT: Child‐Centered Play Therapy
IF: impact factor
MCP: multiple country publications
PTUK: Play Therapy United Kingdom
SCP: single country publications
WoSCC: Web of Science Core Collection

## 1 Introduction

Play therapy initially focused on helping children express emotions and address behavioral problems through nonverbal means, such as toys and role‐play (Elbeltagi et al. 2023). For example, a recent study showed that play therapy can significantly reduce anxious school refusal and other behavioral problems among primary school boys (Shayganfard et al. 2024). As neuroscience and psychological research advance, its theoretical foundations have expanded beyond traditional psychodynamic theory to incorporate brain plasticity theory, which highlights how playful activities foster the reorganization of brain functions (Geertsema et al. 2025; Mindell and Zimmermann 2009).

​​Play therapy is broadly situated within the field of psychotherapy, which encompasses diverse theoretical paradigms, including psychodynamic, humanistic, cognitive‐behavioral, and more recent integrative approaches (Halfon et al. 2016; Roth 2015). Psychodynamic approaches emphasize unconscious processes and early life experiences, while humanistic therapies focus on personal growth and self‐actualization, and cognitive‐behavioral therapy targets maladaptive thoughts and behaviors (Davies et al. 2010; Kenny 2025; Wang et al. 2025). Though often rooted in psychodynamic traditions, play therapy has increasingly integrated elements from these varied frameworks to address emotional, developmental, and behavioral challenges in children (Gillies et al. 2016; Shin et al. 2025).​

Bibliometrics aims to uncover the development trends and knowledge structure in a specific field by quantifying bibliometric indicators such as literature output, citation relationships, and author collaboration networks (Zeng et al. 2024). Visualization tools can also reveal collaborative or co‐occurrence relationships between entities, such as researchers, institutions, countries/regions, and keywords (S. Wang et al. 2025). Two commonly used bibliometric visualization tools are Bibliometrix and VOSviewer, both of which can generate knowledge maps for specific fields (Aria and Cuccurullo 2017; van Eck and Waltman 2010). Bibliometrix offers robust quantitative analyses of publications, citations, and the H‐index. Notably, both tools enable keyword co‐occurrence analysis, and researchers can use them in parallel to cross‐validate the accuracy and consistency of identified research themes and keyword patterns, thereby enhancing the reliability of insights derived from keyword analysis (Li et al. 2024).

Despite the ongoing expansion of clinical practice and theoretical research on play therapy, bibliometric analyses in this field remain scarce. Existing studies have predominantly focused on case reports, clinical trials, or verifying the efficacy of specific treatments, and there is a lack of a systematic, quantitative synthesis of global research findings (Gupta et al. 2023; Xu 2025). Therefore, this study aims to examine the current state of research on play therapy and its evolutionary trajectory using bibliometric methods. By identifying current research hotspots and future frontiers, it seeks to provide new research ideas for researchers in this field.

## 2 Methods

### 2.1 Search Strategy and Inclusion Criteria

Literature on play therapy was retrieved from the Web of Science Core Collection (WoSCC) using a strategy based on medical subject headings (MeSH) and related keywords. The specific search query: TS = (“Play Therapy” OR “Play Therap*” OR “Sandplay Therapy” OR “Sandplay Therap*” OR “Sandplay”). The search string TS = (“Play Therap*”) was selected as a controlled specificity choice. Pilot testing indicated that explicitly adding terms like “Filial Therapy” or “Theraplay” yielded significant overlap and increased dataset noise without improving precision. This strategy ensured high‐quality retrieval while allowing specific sub‐modalities to emerge organically through keyword clustering. The search was limited to the timespan from January 1, 2000, to December 31, 2024, to provide a complete overview of the first 25 years of the 21st century (Table 1). No restrictions were applied regarding language or document type, including articles, reviews, and conference papers, to ensure a comprehensive dataset. Data collection was completed on March 11, 2025, and the study adheres to the PRISMA guidelines 2020 (Sohrabi et al. 2021).

**TABLE 1:** Search strategy.

Item	Search query	Results
**#1**	TS = (“Play Therapy”)	932
**#2**	TS = (“Play Therap*”)	1016
**#3**	TS = (“Sandplay Therapy”)	84
**#4**	TS = (“Sandplay Therap*”)	85
**#5**	TS = (Sandplay)	160
**#6**	#1 OR #2 OR#3 OR #4 OR #5	1168
**#7**	#1 OR #2 OR#3 OR #4 OR #5 Timespan: January 01, 2000, to December 31, 2024	771

### 2.2 Bibliometric Analysis and Visualization Analysis

The research process was conducted independently by two researchers, with any disagreements resolved through consensus. We evaluated several bibliometric indicators, including the number of publications, citations, H‐index, journal impact factor (IF), and JCR category. Publication trends over time were analyzed using linear regression in GraphPad Prism (version 9.0), with the year as the independent variable and annual publication count as the dependent variable. Note that the number of citations was analyzed using all recorded citations, including self‐citations.​

To ensure methodological rigor, the dataset underwent a multi‐stage preprocessing phase. Initial deduplication was performed using automatic tools to eliminate redundant records based on titles and publication years. This was followed by a manual refinement of keywords, in which synonyms, abbreviations, and acronyms were consolidated using standardized terminology such as MeSH to enhance the accuracy of the subsequent thematic analysis. Records lacking critical metadata, such as author affiliations, were excluded from specific sub‐analyses to maintain validity.

### 2.3 Software and Visualization Parameters

We employed Bibliometrix (R version 4.1.3) and VOSviewer (version 1.6.18) to generate multidimensional insights into the scholarly landscape. Within Bibliometrix, we analyzed the productivity and influence of journals, authors, countries/regions, and institutions. For optimal interpretability, visualization parameters were adjusted to highlight the most influential entities, typically focusing on the top 6–10 items. At the same time, longitudinal trends were captured through keyword frequency and “Trend Topics” analyses spanning the entire study period.

Complementary network analyses were performed using VOSviewer to map collaboration networks among authors, institutions, and countries/regions. To ensure visual clarity, these networks were limited to the most interconnected nodes, with a maximum of 1000 entities displayed. For keyword co‐occurrence analysis, we used KeywordPlus terms with a minimum frequency threshold of five occurrences. This approach allowed us to filter out noise and focus the visualization on the field's most significant and recurrent research themes.

### 2.4 Methodological Reflection on Tool Selection

In this study, we deliberately employed a dual‐tool approach, using Bibliometrix (an R package) and VOSviewer, to ensure the comprehensiveness and cross‐validation of our analysis. While both tools can handle literature data, their analytical strengths complement each other. We primarily used Bibliometrix's powerful statistical capabilities to accurately calculate growth rates, the H‐index, and longitudinal “trend topics” based on keyword frequency. VOSviewer was chosen for its superior network visualization algorithms, which are particularly adept at revealing the structural strength of international collaborations and keyword co‐occurrence clusters. This multi‐tool strategy helps improve the overall reliability and granularity of our research results.

## 3 Results

### 3.1 Publications and Citations

**FIGURE 1:**
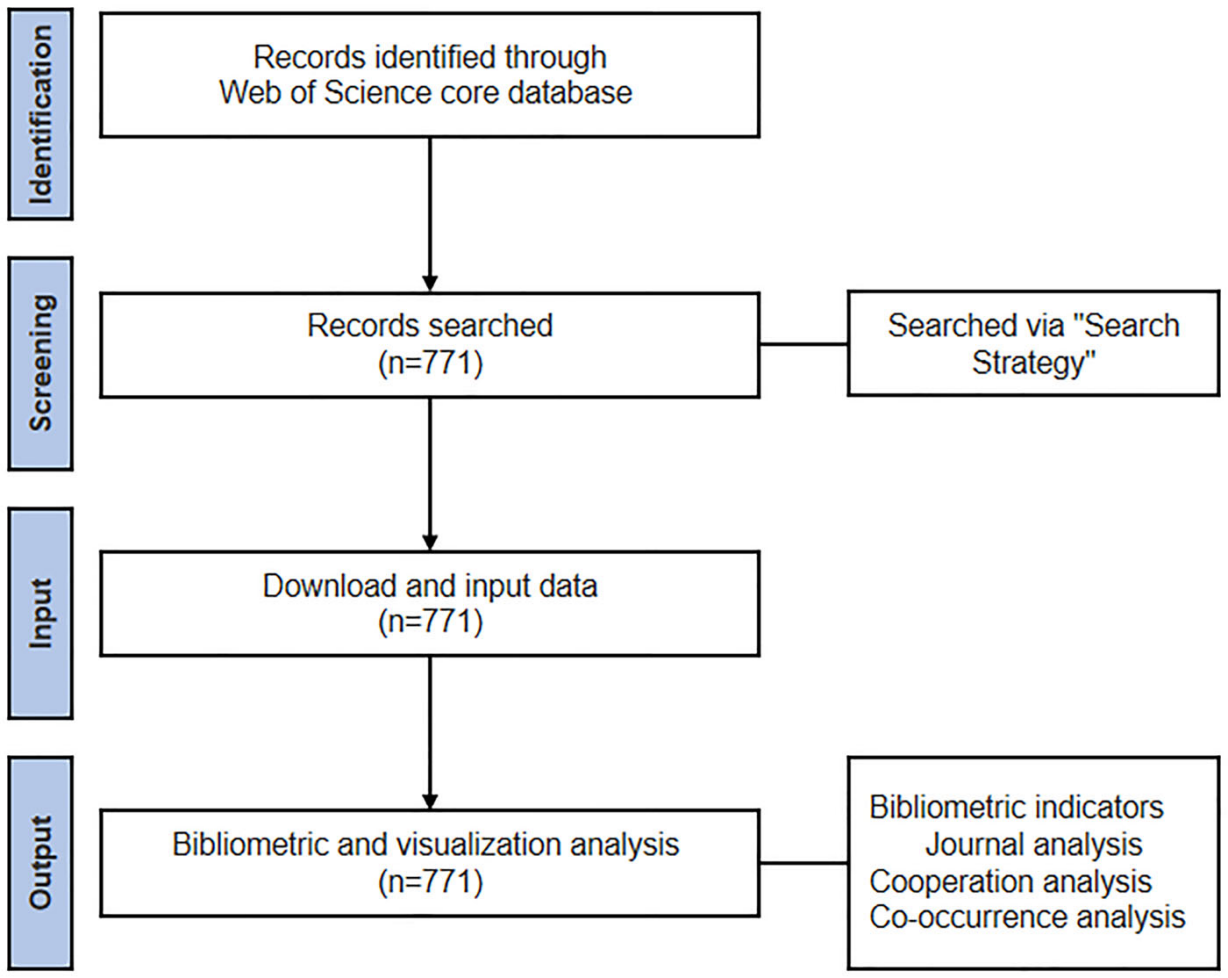
Search flowchart.

**FIGURE 2:**
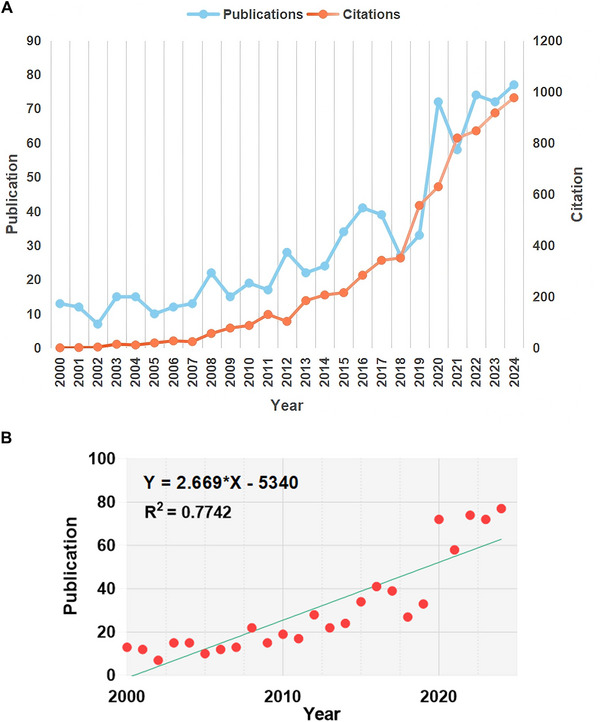
Publications and citations. (A) Line graph of publication and citation. The vertical axis represents publications and citations, and the horizontal axis represents the corresponding years. (B) Linear regression of publication. The vertical axis represents the number of publications, and the horizontal axis represents the year. The equation of the linear regression line is *Y = *2.669 × *X *− 5340. The *R* ^2^ is 0.767. The regression model is used as descriptive trend modeling, not for predictive inference.

The field of play therapy has experienced distinct development phases, with the annual publication volume rising from 13 in 2000 to 77 in 2024 (Figure 1). Notably, a significant inflection point occurred in 2015, after which the growth rate accelerated to 126.5%, reflecting an intensifying global interest (Figure 2A). To descriptively model this overall upward trend, a linear regression was fitted, which showed a significant positive slope (slope = 2.67, 95% CI: 2.05 to 3.29, *R* ^2^ = 0.774, *p *< 0.001) (Figure 2B). It is important to note that this model is presented as a descriptive summary of the trend and was not intended for, nor validated for, predictive inference. While publication peaks were observed in 2012 and 2018, citation peaks did not occur until 2023–2024. This temporal gap suggests that foundational achievements in play therapy require several years of gestation before reaching peak academic influence. Furthermore, the tripling of average citations per article (4.3 to 12.7) underscores the field's transition from niche exploration to a high‐impact clinical discipline.

### 3.2 Journals Analysis

**FIGURE 3:**
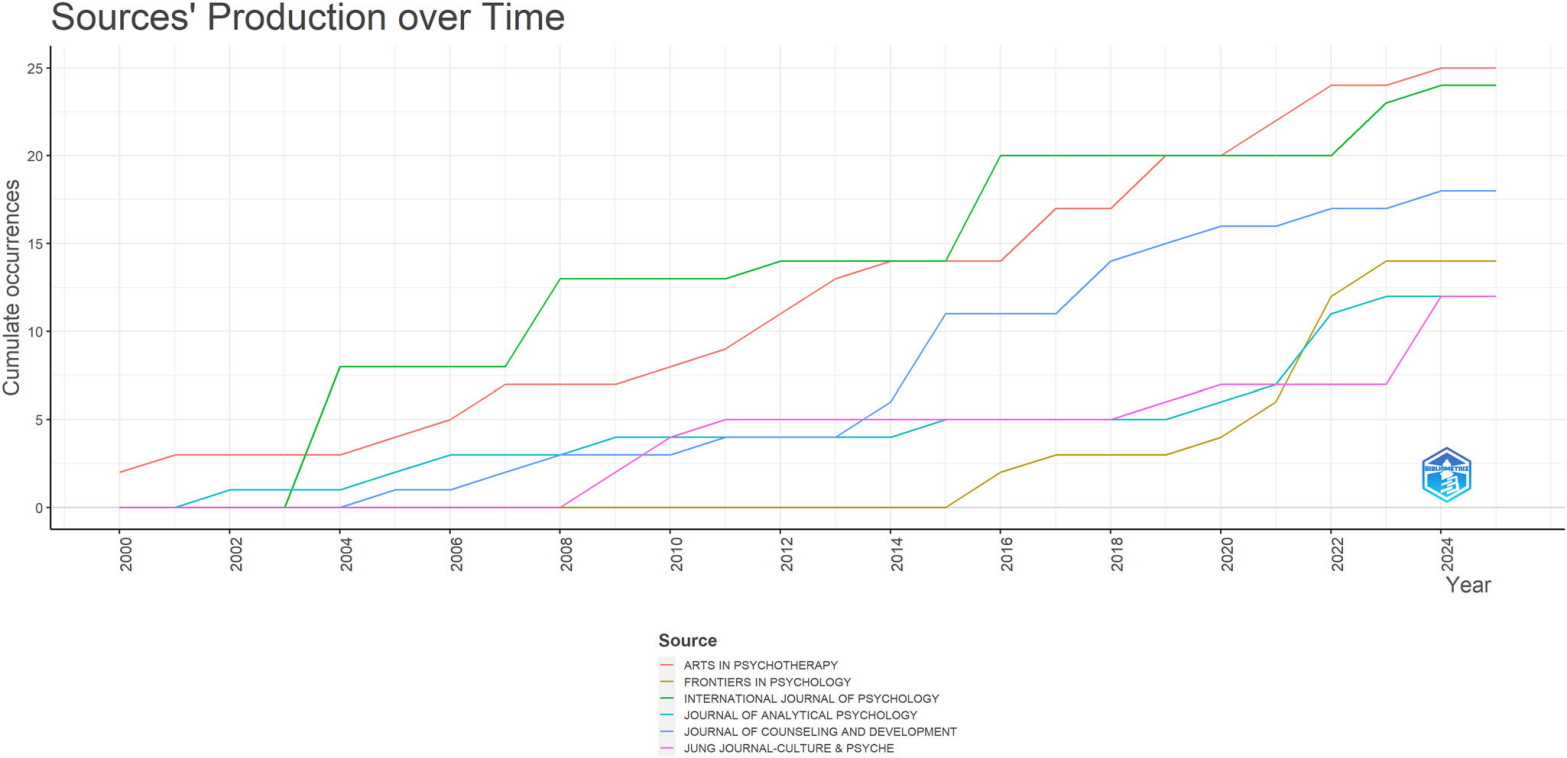
Production of journals. The vertical axis represents publications, and the horizontal axis represents the corresponding years.

Journal productivity reveals a shifting landscape of influence (Table S1). While the *Arts in Psychotherapy* and the *International Journal of Psychology* have historically led in volume, their dominance has been cyclical. The *Arts in Psychotherapy* reclaimed the top position in 2021 after a long period of leadership by the *International Journal of Psychology* (Figure 3). A key insight is that the *Journal of Clinical Child and Adolescent Psychology*, despite a low publication volume (*n* = 4), has the highest average citation rate (109.75). This indicates that the journal acts as a premium outlet for high‐impact clinical evidence, prioritizing quality and rigorous validation over quantity.

### 3.3 Authors

A total of 2305 authors published these articles in this field, and we further list the bibliometric indicators of the top 10 authors (Table S3). Among them, Ray D.C. had the most published articles (12), followed by Halfon S. (7), Bratton S.C. (7), and Baggerly J. (6). In addition, Ray D.C. also had the highest H‐index (7). Figure S1 shows the variation in the authors’ output over time. Bratton S.C. has the highest citation volume each year. Ray D.C., Bratton S.C., and Ceballos Peggy all published three articles in a year: 2015, 2014, and 2022, respectively. Meanwhile, Rousseau Cecile had the longest research period, from 2007 to 2024, totaling 18 years. The most prolific author in recent years is Brown Ted, who published two articles in each of 2022 and 2023.

### 3.4 Countries/Regions and Institutions

The United States has the most published articles, with 228 (Table S3). Of these, 208 were produced by a single country/region, and 20 were developed through multicountry collaboration. With 95 published articles, China ranked second, of which 11 articles were published through multicountry collaboration, accounting for 11.6%. With seven publications on international collaboration and 35 on domestic output, Spain ranked third overall. The top three countries by citations were the United States (*n* = 3092), China (*n* = 1118), and the United Kingdom (*n* = 677). In addition, the top three institutions by the number of published articles were all from the United States. They are the University of North Texas, Harvard University, and the State University System of Florida, in that order (Table S4).

### 3.5 Collaborations

**FIGURE 4:**
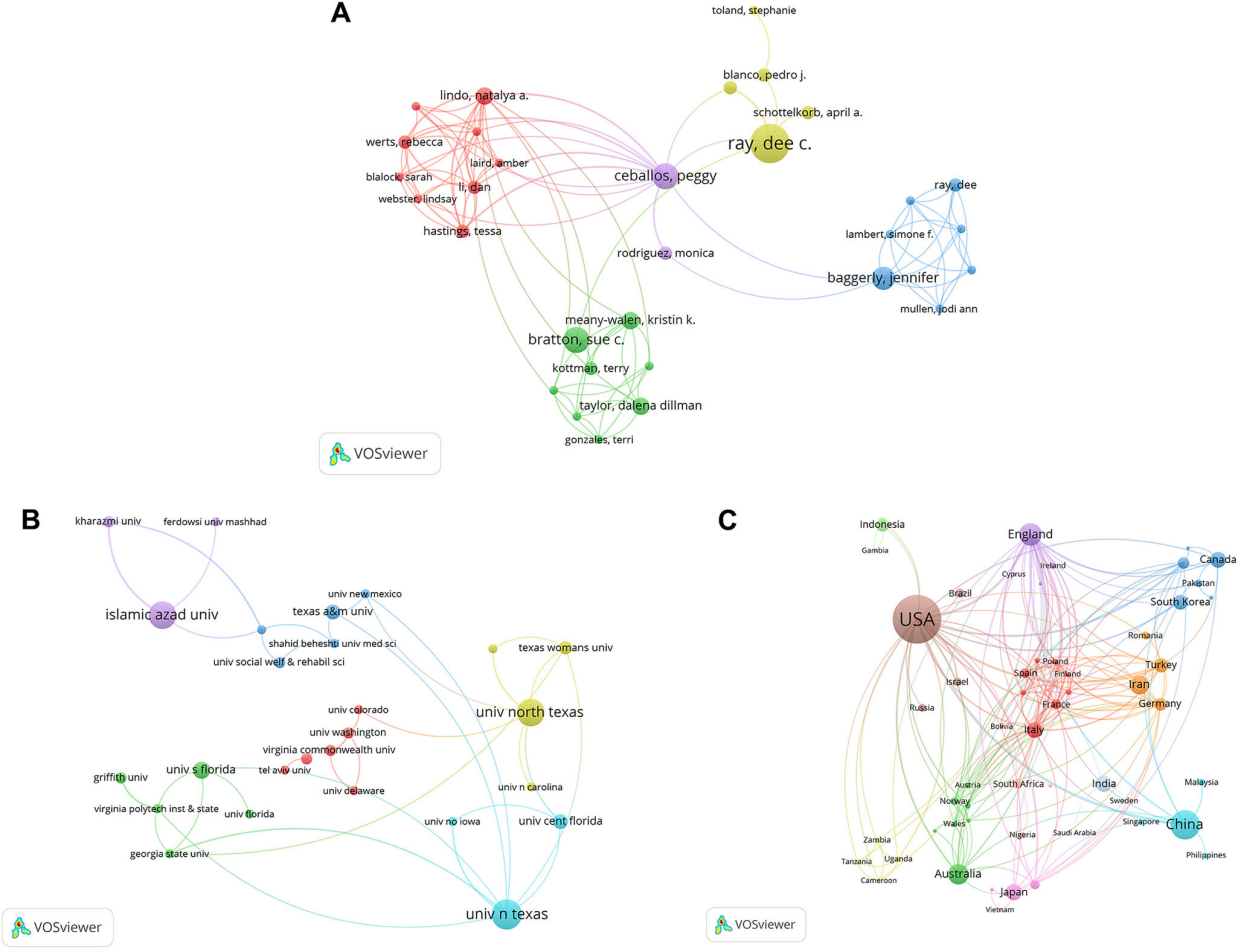
Collaboration situation between authors (A), institutions (B), and countries/regions (C). A node represents an individual (author, country/region, or institution), and nodes of the same color belong to the same cluster. The links connecting nodes represent the collaborative relationships between them.

We further summarized the collaboration among individuals (Figure 4). Author collaboration is divided into five clusters (Figure 4A). The author who collaborated most frequently with others was Halfon Sibel, followed by Ceballos Peggy and Lindo Natalya A. Figure 4B shows collaboration among countries. In addition to having the most citations and publications, the United States also had the most international collaboration. Italy and the United Kingdom were the countries/regions that engaged in the most international collaboration after the United States. Notably, although China ranked second in the number of publications, it was not among the top three in international collaboration. Moreover, Figure 4C shows the collaboration among institutions. The University of North Texas collaborates most closely with other institutions, while London's Global University and Harvard University have many collaborations with other institutions.

### 3.6 Keywords

**FIGURE 5:**
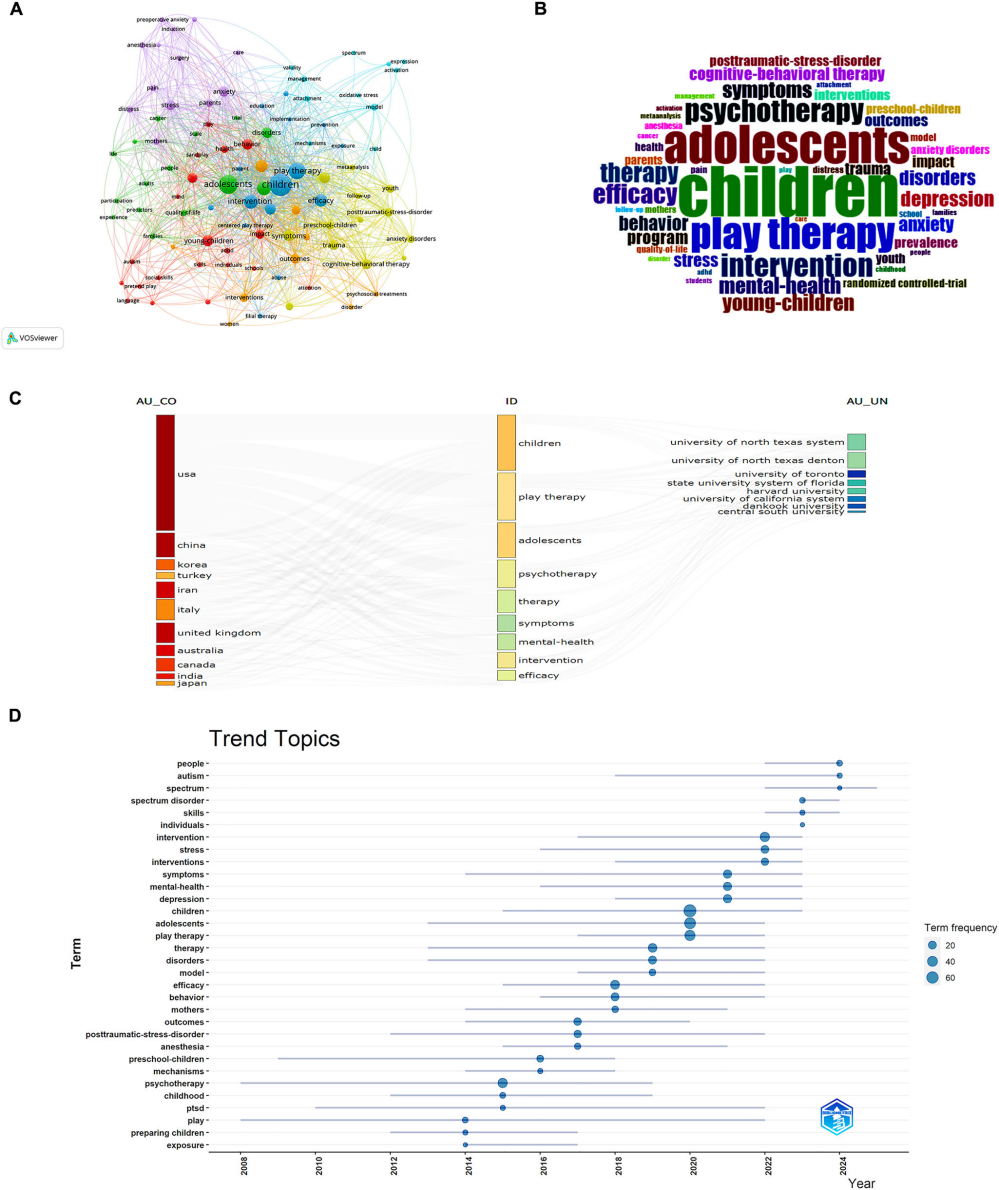
Comprehensive keyword analysis and research trends. (A) Keyword co‐occurrence network map (via VOSviewer). Node represents a keyword, with its size proportional to the occurrence frequency. Connecting lines indicate co‐occurrence relationships. The network highlights primary research keywords and their interconnections.​ (B) Keyword cloud (via Bibliometrix). Font size reflects keyword frequency, visually emphasizing the most frequently occurring terms in the dataset.​ (C) Three‐field plot. This visualization illustrates the flow and thematic connections between countries/regions (left), keywords (middle), and institutions (right), highlighting the geographic and institutional focus of the research.​ (D) Topic trend analysis. This plot identifies the emergence and duration of specific research topics over time, reflecting the strategic shifts and frontiers in the field's knowledge focus.

The top three keywords with the highest frequency of occurrence are “children” (*n* = 70), “adolescents” (*n* = 53), and “play therapy” (*n* = 44) (Table S5 and Figure 5A,B). The results obtained by the two visualization tools were consistent. We further analyzed the annual changes in keywords. The United States has conducted research on fields related to these keywords. The institution with the broadest coverage of the keywords was the University of North Texas (Figure 5C). Except for psychotherapy, which briefly exceeded the frequency of children in 2008 and 2009, the frequency of children was highest in all other years (Figure S2). Moreover, trend analysis revealed that the top three keywords by appearance rate all experienced explosive growth in 2020. The trend topics for 2024 were “people,” “autism,” and “spectrum” (Figure 5D).

## 4 Discussion

The evolution of play therapy literature demonstrates significant phased growth, reflecting the field's expanding academic influence and its maturation as a clinical discipline. Our analysis reveals a characteristic 1‐ to 2‐year lag between publication and citation peaks, highlighting the typical time delay in the dissemination and integration of academic achievements. Historically, *Arts in Psychotherapy* and the *International Journal of Psychology* have served as core carriers, reflecting the field's foundational reliance on psychological methodology. However, the most striking impact of the *Journal of Clinical Child and Adolescent Psychology* signals a critical shift. From a policy and practice perspective, this shift indicates that play therapy is moving beyond theoretical validation toward “evidence‐based practice” (Lin and Bratton 2015; Parker et al. 2021). For practitioners, this necessitates a focus on standardized, measurable interventions. At the same time, for policymakers, such rigorous data provide the empirical foundation required to include play therapy in national health guidelines and insurance reimbursement frameworks.

The influence of individual scholars mirrors this shifting focus. The high annual citation volume of authors like Bratton S.C. and Ray D.C. highlights the enduring impact of child‐centered approaches (Cornett and Bratton 2014; Meany‐Walen et al. 2014; Ojiambo and Bratton 2014; Ray et al. 2015; Stulmaker and Ray 2015; Ware Balch and Ray 2015). While sporadic productivity bursts from scholars Ceballos Peggy may stem from increased funding (Baggerly et al. 2022; Lindo et al. 2022; Ray et al. 2022), they function as vital catalysts for subsequent theoretical and empirical advancements in clinical practice. This dominance, however, must be understood within the context of professional credentialing. The field is heavily shaped by US and UK institutions such as the Association for Play Therapy (APT) and Play Therapy United Kingdom (PTUK). Their accreditation guidelines heavily influence publication topics and preferred modalities, specifically non‐directive Child‐Centered Play Therapy (CCPT) and Filial Therapy. Thus, the centrality of figures like Landreth, Bratton, and Ray in our co‐citation networks reflects these institutional frameworks rather than being a purely neutral academic observation. These scholars have acted as the primary architects of the pedagogical models required for professional certification.

The United States continues to exert dominant influence in play therapy research, a position rooted in its historical role as the cradle of the field and reinforced by a mature infrastructure for professional certification, clinical training, and sustained research funding. Since Virginia Axline's pioneering development of CCPT, grounded in humanistic principles (Landreth 1993), US‐based institutions have remained central to theoretical innovation and knowledge production. This concentrated influence fosters a systemic bias, framing Western developmental models as universal despite their limited resonance in Global South contexts where communal values dictate play behavior (Akhtar et al. 2025). Emerging evidence from the Global South challenges this assumed universality and underscores the importance of prioritizing cultural adaptation over simple adoption. A recent meta‐analysis of the Global South demonstrates that play therapy yields significant effect sizes in reducing childhood aggression (Alfath et al. 2025); similarly, empirical work in the Philippines confirms the efficacy of short‐term, resource‐efficient models adapted for limited clinical infrastructure (Bengwasan 2023). Disaster‐response frameworks in Puerto Rico and Indonesia illustrate play therapy's evolution into communal and family‐centered contexts, shifting healing from the individual to the broader social ecology (Nilsson et al. 2024). Collectively, these cases suggest that the true universality of play therapy does not reside in rigid adherence to Western protocols, but in the modality's capacity to be reconfigured in response to diverse cultural, structural, and systemic realities.

The keyword clusters identified in this study, ranging from “children” and “intervention” to “attachment” and “play behavior,” reflect a sophisticated evolution of theoretical paradigms. Historically, the field has been anchored in Humanistic traditions, evidenced by the persistent dominance of “child‐centered” and “relationship” themes (Alemdar and Karaca 2025). We find that the prominence of “attachment” and “internalizing problems” signals a robust Psychodynamic foundation, which is increasingly bridged with Cognitive Behavioral Therapy (CBT) paradigms, as reflected in terms such as “efficacy,” “symptoms,” and “intervention.” This suggests that the modern landscape of play therapy is moving away from isolated theoretical silos toward a multi‐theoretic synthesis. This transition toward an integrative, neurodiversity‐informed framework, as evidenced by the clustering of “autism” with “evidence‐based” keywords, demonstrates how the field is recalibrating its humanistic roots to address increasingly complex clinical symptoms. Furthermore, the dominance of these topics suggests that the relevant literature is primarily influenced by Western developmental psychology frameworks that prioritize individual autonomy and emotional self‐regulation (Henrich et al. 2010).

Collaboration patterns in play therapy research reveal differentiated dynamics across author, national/regional, and institutional levels, reflecting both productive synergies and persistent structural inequities. At the author level, network hubs such as Halfon Sibel play a key role in facilitating cross‐disciplinary knowledge exchange. At the national and regional levels, the United States continues to dominate in both research output and international collaboration. In contrast, other high‐output countries, such as China, remain less integrated into global networks. Institutional collaborations, exemplified by the University of North Texas, Harvard University, and University College London, further demonstrate how concentrated resources, funding, and expertise can elevate research quality. However, this concentration of influence is not solely a function of productivity; it is closely embedded within institutional accreditation and funding ecosystems. Organizations such as the Association for the APT and PTUK act as gatekeepers to the field through credentialing systems that require specialized training and supervision, predominantly anchored in high‐output Western institutions. As a result, these hubs benefit from a self‐reinforcing cycle in which they attract funding, international doctoral students, and epistemic authority, thereby defining dominant clinical paradigms, such as CCPT. This centralized structure has significant implications for global equity, as researchers from the Global South are often relegated to peripheral roles or required to align with Northern theoretical frameworks to participate in international collaborations. “Global” collaboration often represents the geographic expansion of Western models rather than mutual exchange, thereby marginalizing indigenous practices and limiting the research's inclusivity (Akhtar et al. 2025; Francis et al. 2022; Jude et al. 2025).

Research hotspots have increasingly converged on children and adolescents, with a modern emphasis on validating the efficacy of interventions for neurodevelopmental challenges. Among the high‐frequency keywords, “children,” “adolescents,” and “young children” indicate that children and adolescents are the core research subjects in this field, and the research conducted on them is extensive and in‐depth (Bartakke et al. 2025; Huang et al. 2024; Shrimahalakshmi et al. 2025). “Intervention” and “efficacy” reflect the research's focus on intervention methods and the evaluation of treatment effects, whereas “mental health” and “symptoms” focus on mental health and symptom improvement (Adarsh et al. 2022; Koukourikos et al. 2021). The surge in keywords such as “autism” and “spectrum” since 2024 indicates the expansion of play therapy into neurodiversity‐informed care (Camino‐Alarcón et al. 2024; Wadley and Stagnitti 2024). Recent advances in understanding the neurobiological underpinnings of autism spectrum disorder, alongside increased emphasis on early intervention and personalized therapeutic strategies, have likely contributed to the growing academic attention to the use of play therapy in this population (Davidson and Stagnitti 2021; Sun et al. 2025). Clinically, this requires therapists to integrate neurobiological insights into their work, moving toward relationship‐based, non‐directive interventions supporting neurodevelopmental plasticity (Jamei et al. 2024; Metlek and Çağlar 2024). Rather than focusing on extinguishing atypical behaviors, practitioners should leverage play therapy's non‐directive roots to support neurodevelopmental plasticity and emotional regulation. This involves creating sensory‐friendly therapeutic environments and integrating strength‐based approaches that respect the unique communicative styles of neurodivergent children. For policymakers, this supports the integration of play therapy into early intervention public programs, improving long‐term outcomes and reducing lifelong support of burdens for neurodivergent populations (Bate et al. 2019; Kollbrunner and Seifert 2013; Lee et al. 2023). The transdiagnostic efficacy of play‐based interventions across the lifespan, from early autism diagnosis to geriatric Alzheimer's care, suggests that funding should be redirected toward multidisciplinary care settings. Specifically, policymakers should advocate for the inclusion of play therapy in insurance reimbursement schemes and public health mandates, recognizing it as a cost‐effective, evidence‐based strategy to enhance long‐term functional outcomes and reduce the economic burden on lifelong disability support systems.

The emergence of “people” as a trend word signals a paradigm shift toward a lifespan perspective (Jamei et al. 2024). The application of play therapy to fathers with postpartum depression and elderly individuals with Alzheimer's disease demonstrates its transdiagnostic potential (Ogawa et al. 2016; Vaccaro et al. 2020; You et al. 2025). Integrating play therapy into paternal interventions significantly reduces postpartum depressive symptoms and mitigates the intergenerational transmission of mental health risks (Husain et al. 2025). In addition, a meta‐analysis revealed that play therapy can significantly improve agitation behavior, apathy, and psychological state of Alzheimer's disease patients (Peng et al. 2024). These developments suggest that play therapy is increasingly viewed not merely as a child‐focused modality, but as a versatile, person‐centered approach with transdiagnostic potential. Further research is needed to explore its differential effectiveness and mechanisms of action across varied demographic and clinical groups.

While we define “play therapy” broadly, it is essential to distinguish between psychotherapeutic approaches, such as CCPT, therapeutic play in medical settings, and developmental play. We must also acknowledge that bibliometric clustering tends to blur these nuanced theoretical distinctions. Because keyword indexing in databases like WoSCC can be inconsistent, diverse practices are often aggregated. This methodological reflection is crucial for maintaining theoretical rigor and ensuring that specific therapeutic mechanisms are not overlooked. Moreover, while providing a robust macro‐perspective, this bibliometric analysis underrepresents family‐embedded and community‐based interventions prevalent in the Global South, which are often published in regional outlets not indexed by the WoSCC. This geographic and linguistic indexing bias suggests that the apparent dominance of clinic‐based, individualistic models reflects the “visible” scholarly landscape within major databases rather than the whole global spectrum of play‐based initiatives. Consequently, these findings must be interpreted with caution, acknowledging the invaluable yet less visible work occurring across diverse regional and collectivist contexts to ensure a balanced understanding of the field's true diversity.

Compared with earlier reviews that primarily documented publication trends, dominant modalities, or geographic distributions in play therapy (Peng et al. 2024; Ulaş, Seçer, Victory, & McNeil, 2023), the present findings point to deeper theoretical and structural implications for the field. Notably, a paradigm shift occurred between 2020 and 2024, transitioning from traditional humanistic, relationship‐based models toward integrative, trauma‐informed, and neurodiversity‐oriented frameworks. Importantly, this shift does not occur in a neutral knowledge landscape. By examining patterns of authorship, institutional affiliation, and indexing, the study highlights a persistent visibility gap in which Global South scholarship remains underrepresented. Rather than reflecting differences in research quality alone, this imbalance appears to be shaped by institutional credentialing systems, such as APT and PTUK, and database indexing practices, which systematically privilege Anglophone and US‐based research. Contextually grounded adaptations in Indonesia and the Philippines necessitate a re‐evaluation of how “mainstream” knowledge is defined and whose contributions are recognized in the global literature.

Finally, from a theoretical perspective, the evolving application of play therapy, particularly in relation to autism spectrum disorder, neurodegenerative conditions, and transdiagnostic frameworks, invites deeper integration with neuroscience. Understanding the neural mechanisms underlying emotional regulation, attachment, and developmental plasticity can provide valuable insights into why and how play‐based interventions exert their effects (Connaughton et al. 2025; Money et al. 2021; Stewart et al. 2016; X. Wang et al. 2025; Willis et al. 2014). This expansion suggests a holistic public health strategy that integrates play therapy into multidisciplinary care settings, such as geriatric units and maternity clinics. As the modality expands to all age groups, practitioners must remain adaptable, designing individualized intervention plans that account for varied demographic and clinical needs. Engaging with culturally grounded practice is essential to ensure that interventions are not only evidence‐based but also culturally resonant. Integrating clinical practice with developmental neuroscience research will solidify the scientific foundation of play therapy within the evolving healthcare landscape.

### 4.1 Limitations and Prospects

There are several limitations in this study. First, our data are restricted to the WoSCC database, which primarily indexes high‐impact, English‐language journals. This creates a “visibility gap,” potentially excluding relevant studies from regional, non‐English journals, grey literature, and unpublished research from the Global South (Fei et al. 2025).​ Future studies should incorporate Scopus and PubMed to enhance cross‐database inclusiveness.

Second, while our analysis identifies central hubs in the collaboration network, the structural drivers behind this dominance, such as the influence of Western accreditation systems, including APT and PTUK, and funding availability, require more granular investigation. These institutional factors often dictate research agendas and may skew international collaboration toward the “geographic extension” of Northern models rather than a mutual cultural exchange (Liu et al. 2024).

Furthermore, a notable methodological limitation is the risk that keyword clustering may blur the distinctions among psychotherapeutic play therapy, therapeutic play in medical settings, and developmental play. Because database indexing often aggregates these heterogeneous practices under broad terms, our macro‐perspective may overlook nuanced therapeutic mechanisms unique to clinical settings. To address this, future research should utilize ontology‐based text mining or manual content coding on specialized datasets to distinguish the “corrective” functions of clinical play therapy from broader “supportive” developmental frameworks.

Finally, because bibliometric methods rely on published retrospective data, they inevitably lag behind the most recent unpublished innovations and ongoing projects (Yang et al. 2025). Continuous updates and the integration of qualitative interviews with leading scholars would help capture the field's real‐time trajectory and ensure a more inclusive understanding of play therapy's global evolution.

## 5 Conclusion

In summary, play therapy has transitioned from a specialized clinical practice into a sophisticated, evidence‐based global discipline. While US and UK institutional frameworks currently dictate the research landscape and pedagogical standards, the long‐term vitality of the field depends on challenging the Western‐centric “gold standard” by amplifying community‐embedded models and cultural adaptations from the Global South. Thematic shifts indicate a profound conceptual synthesis, as the discipline's humanistic roots increasingly converge with trauma‐informed and neurodiversity‐focused paradigms. As play therapy extends its transdiagnostic reach across the lifespan, from early childhood neurorehabilitation to geriatric care, future advancements must harmonize the precision of developmental neuroscience with a deep commitment to sociocultural equity. By dismantling structural barriers to publication visibility and fostering more inclusive international partnerships, the play therapy community can ensure that these interventions remain both scientifically rigorous and culturally resonant for children and families worldwide.

## Author Contributions

Conception and design of the study: Yaqian Gao and Yue Hu. Acquisition and analysis of data: Xinxing Fei, Shiqi Wang, Jianxiong Wang, and Yaqian Gao. Drafting the figures: Xinxing Fei and Shiqi Wang. Drafting of the manuscript: All co‐authors. Critical revision of the manuscript: All co‐authors. Approval of the final version for publication: All co‐authors.

## Funding

The authors have nothing to report.

## Conflicts of Interest

The authors declare no conflicts of interest.

## Supporting information




**Supplementary Materials**: brb371301‐sup‐0001‐SuppMat.pdf

## Data Availability

The source data are from WoSCC. The datasets used and/or analyzed during the current study are available from the corresponding author on reasonable request.
