# Supplementary material for: Knowledge Structure and Evolution of Hotspots in Play Therapy: A Bibliometric Analysis via Multiple Visualization Tools
Source: Brain Behav. 2026 Mar 10;16(3):e71301. doi: 10.1002/brb3.71301 (PMC12973151; doi:10.1002/brb3.71301)
Supplement: Supplementary file 1 — Supplementary Materials: brb371301‐sup‐0001‐SuppMat.pdf [file BRB3-16-e71301-s001.pdf]

Supplementary Material

Table S1 The top 10 journals in terms of scientific productivity

Rank	Journal	Publication	IF	JCR
1	*Arts in Psychotherapy*	25	1.5	Q3
2	*International Journal of Psychology*	24	3.3	Q1
3	*Journal of Counseling and Development*	18	2.3	Q2
4	*Frontiers in Psychology*	14	2.6	Q2
5	*Journal of Analytical Psychology*	12	0.5	Q2
6	*Jung Journal-Culture & Psyche*	12	0.1	-
7	*Clinical Social Work Journal*	8	2.7	Q1
8	*American Journal of Family Therapy*	7	0.9	Q3
9	*Children And Youth Services Review*	7	2.4	Q1
10	*International Journal of Play*	7	0.6	Q3

IF: impact factor.

Table S2 The top 10 authors in terms of scientific productivity

Rank	Author	Publication	H-index	G-index	M-index	Citation	Year of first publication
1	Ray DC	12	7	12	0.389	202	2008
2	Bratton SC	7	7	7	0.333	472	2005
3	Halfon S	7	5	7	0.5	68	2016
4	Baggerly J	6	5	6	0.238	98	2005
5	Rousseau C	5	4	5	0.211	115	2007
6	Kim M	4	3	4	0.333	26	2017
7	Cohen D	3	3	3	0.273	218	2015
8	Daniel S	3	3	3	0.429	24	2019
9	Kil T	3	3	3	0.429	22	2019
10	Lacroix L	3	3	3	0.158	83	2007

Table S3 The top 10 countries/regions in terms of scientific productivity

Rank	Country	Publication	SCP	MCP	MCP %	Citation
1	USA	228	208	20	8.8	3092
2	China	95	84	11	11.6	1118
3	England	42	35	7	16.7	677
4	Australia	40	37	3	7.5	338
5	Iran	32	30	2	6.3	51
6	India	25	23	2	8	163
7	Canada	23	19	4	17.4	340
8	Korea	21	17	4	19	122
9	Indonesia	19	17	2	10.5	24
10	Japan	18	14	4	22.2	55

MCP: multiple country publications; SCP: single country publications.

Table S4 The top 10 institutions in terms of scientific productivity

**Rank**	**Institution**	**Publication**
1	University of North Texas	35
2	Harvard University	24
3	State University System of Florida	22
4	Islamic Azad University	21
5	Central South University	20
6	University Of California System	17
7	Dankook University	16
8	University Of Toronto	16
9	University Of London	15
10	Beijing Normal University	14

Table S5 The top 10 keywords

Rank	Keywords	Occurrences
1	children	70
2	adolescents	53
3	play therapy	44
4	intervention	33
5	psychotherapy	32
6	efficacy	28
7	therapy	27
8	mental-health	23
9	symptoms	23
10	young-children	23

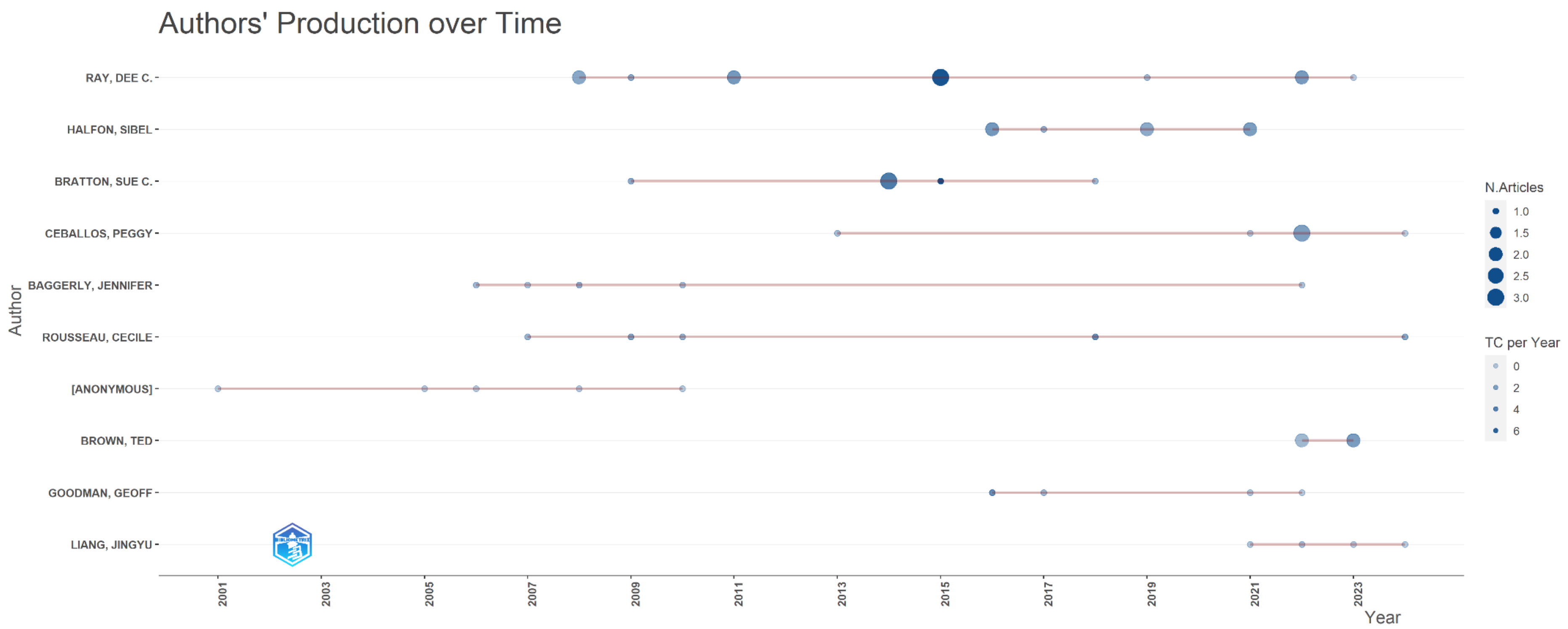

Figure S1 Production of authors. The horizontal axis represents the year of publication. The size of the circle represents the number of publications; the larger the circle is, the greater the number of publications. The color of the circle represents the total annual citation volume. The larger the citation volume is, the darker the color. The length of the red line represents the duration of the research. The longer the red line is, the longer the research time.

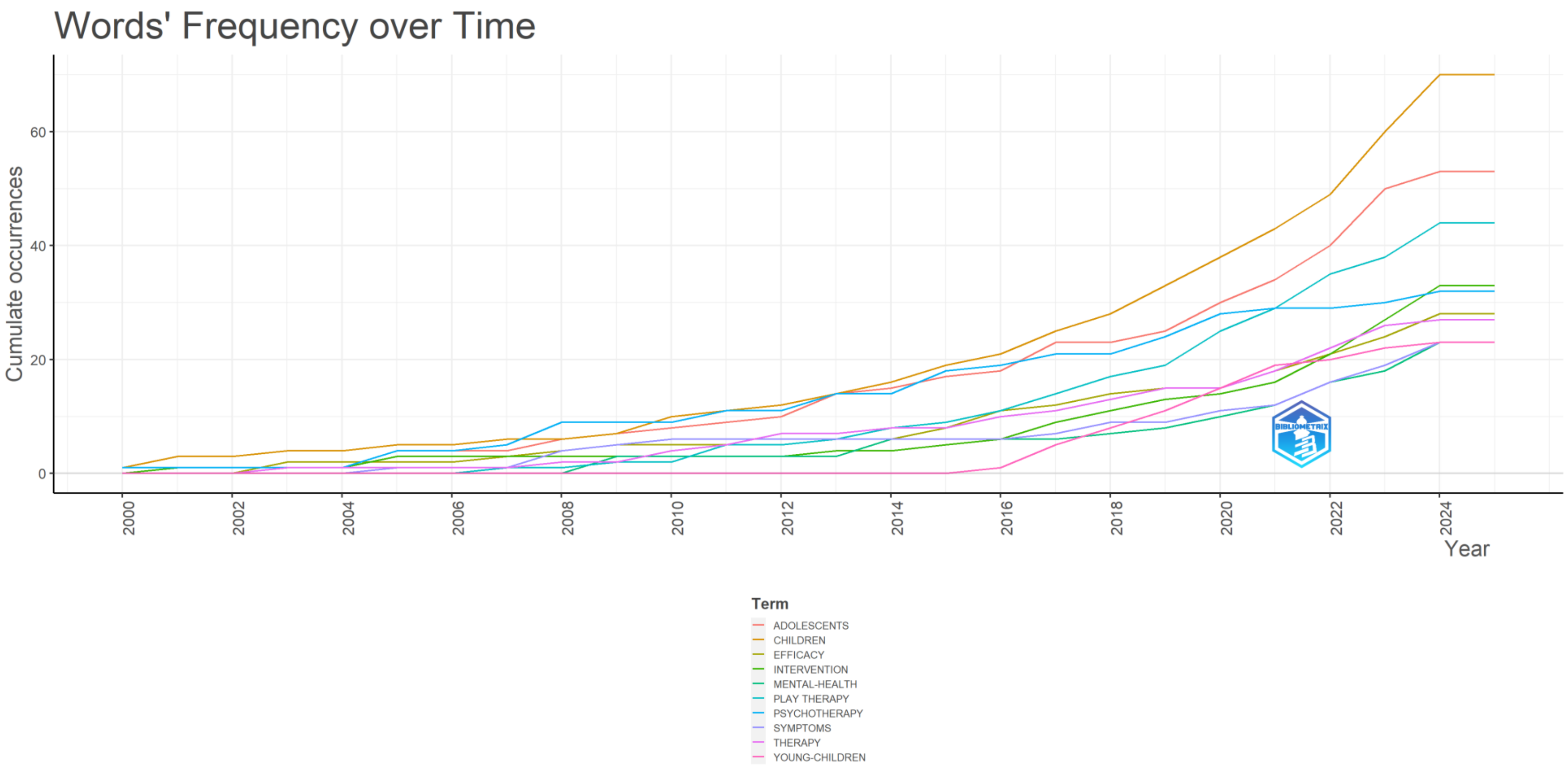

Figure S2 Keyword timezone view. This map displays the evolution of keywords over time. The vertical axis represents the frequency of keywords, and the horizontal axis represents the corresponding years, revealing the temporal progression of the knowledge structure.
